# Impact of Silver and Iron Nanoparticle Exposure on Cholesterol Uptake by Macrophages

**DOI:** 10.1155/2015/127235

**Published:** 2015

**Authors:** Jonathan H. Shannahan, Hari Sowrirajan, Indushekhar Persaud, Ramakrishna Podila, Jared M. Brown

**Affiliations:** 1 Department of Pharmaceutical Sciences, Skaggs School of Pharmacy and Pharmaceutical Sciences, The University of Colorado Anschutz Medical Campus, Aurora, CO 80045, USA; 2 Cherry Creek High School, Greenwood Village, CO 80111, USA; 3 Department of Physics and Astronomy, Clemson University, Clemson, SC 29634, USA; 4 Clemson Nanomaterials Center and COMSET, Clemson University, Anderson, SC 29625, USA

## Abstract

Macrophages are central to the development of atherosclerosis by absorbing lipids, promoting inflammation, and increasing plaque deposition. Nanoparticles (NPs) are becoming increasingly common in biomedical applications thereby increasing exposure to the immune and vascular systems. This project investigated the influence of NPs on macrophage function and specifically cholesterol uptake. Macrophages were exposed to 20 nm silver NPs (AgNPs), 110 nm AgNPs, or 20 nm Fe_3_O_4_NPs for 2 h and NP uptake, cytotoxicity, and subsequent uptake of fluorescently labeled cholesterol were assessed. Macrophage uptake of NPs did not induce cytotoxicity at concentrations utilized (25 *μ*g/mL); however, macrophage exposure to 20 nm AgNPs reduced subsequent uptake of cholesterol. Further, we assessed the impact of a cholesterol-rich environment on macrophage function following NP exposure. In these sets of experiments, macrophages internalized NPs, exhibited no cytotoxicity, and altered cholesterol uptake. Alterations in the expression of scavenger receptor-B1 following NP exposure, which likely influences cholesterol uptake, were observed. Overall, NPs alter cholesterol uptake, which may have implications in the progression of vascular or immune mediated diseases. Therefore, for the safe development of NPs for biomedical applications, it is necessary to understand their impact on cellular function and biological interactions in underlying disease environments.

## 1. Introduction

Nanotechnology is a rapidly expanding field that is transforming numerous areas of technology including a variety of biomedical applications. Specifically, through the development of unique nanoparticles (NPs) there has been the expansion of various drug delivery platforms. Two particular NPs, which have gained interest for a variety of biomedical applications, include silver NPs (AgNPs) and iron oxide NPs (Fe_3_O_4_ NPs). AgNPs are increasingly being utilized due to their antimicrobial properties and have been incorporated in products such as textiles, household appliances, food storage containers, and medical devices such as i.v. catheters and lines [1–4]. Fe_3_O_4_ NPs have been proposed as drug delivery platforms and for their use as magnetic resonance imaging contrast agents [5–7]. Although NPs are increasingly being incorporated into every aspect of our society, we currently lack sufficient knowledge regarding their toxicity. Specifically, AgNPs have been shown to induce a variety of toxic responses including oxidative stress, inflammatory responses, apoptosis, and cytotoxicity in a variety of different cell types [8–12]. Investigation regarding the toxicity of Fe_3_O_4_ NPs has demonstrated limited toxicity in terms of no observed genotoxicity and minor cytotoxicity at high concentrations (>100 *μ*g/mL) [13, 14].

Additional research is also needed to understand how NP exposures can modify normal cell function at concentrations that do not elicit overt cytotoxicity. Furthermore, few studies have evaluated the influence of underlying disease states on NP-induced toxicity or the influence of NP exposure on progression and development of disease states. Individuals with underlying cardiovascular disease and/or obesity comprise a significant and growing portion of the population. *In vivo* animal studies have demonstrated that these individuals may be increasingly sensitive to toxicological insults [15–17]. To more accurately screen NPs for toxicity, it is necessary to understand how common underlying disease states alter the cellular environments (such as high cholesterol), modify NP function, and alter biological responses.

A disease of primary concern for our population is atherosclerosis. The development of atherosclerosis is mediated by macrophage uptake of cholesterol within artery walls leading to inflammation and the formation of an atherosclerotic plaque. Due to their location and immune surveillance properties, macrophages are likely one of the first cell types to interact with NPs when introduced into the circulation mediating their clearance. Macrophages interact with both cholesterol and NPs through scavenger receptors expression on their surface [18–20]. Scavenger receptors are pattern recognition receptors that are classified into three types: Class A, Class B, and Class C. These receptors recognize a number of ligands including oxidized-lipoproteins, pathogens, and negatively charged foreign particles such as NPs [18, 21]. Association of ligands with scavenger receptors facilitates cellular uptake, ligand removal, and proinflammatory responses [12, 22]. Further scavenger receptors are known to be involved in lipid metabolism as well as atherosclerosis development [23]. Specifically, AgNPs interact with scavenger receptors on the surface of macrophages thus facilitating uptake and apoptosis [24]. Previously, we have demonstrated that inhibition of scavenger receptor-B1 (SR-B1) can reduce the uptake of AgNPs by rat aortic endothelial cells as well as reduce AgNP-induced cytotoxicity and inflammatory response [12]. Mice deficient in SR-B1 have demonstrated increased levels of plasma cholesterol compared to wild-type [23]. This finding as well as the high affinity of SR-B1 for lipoproteins suggests a critical role for SR-B1 in lipoprotein metabolism.

**Figure 1:**
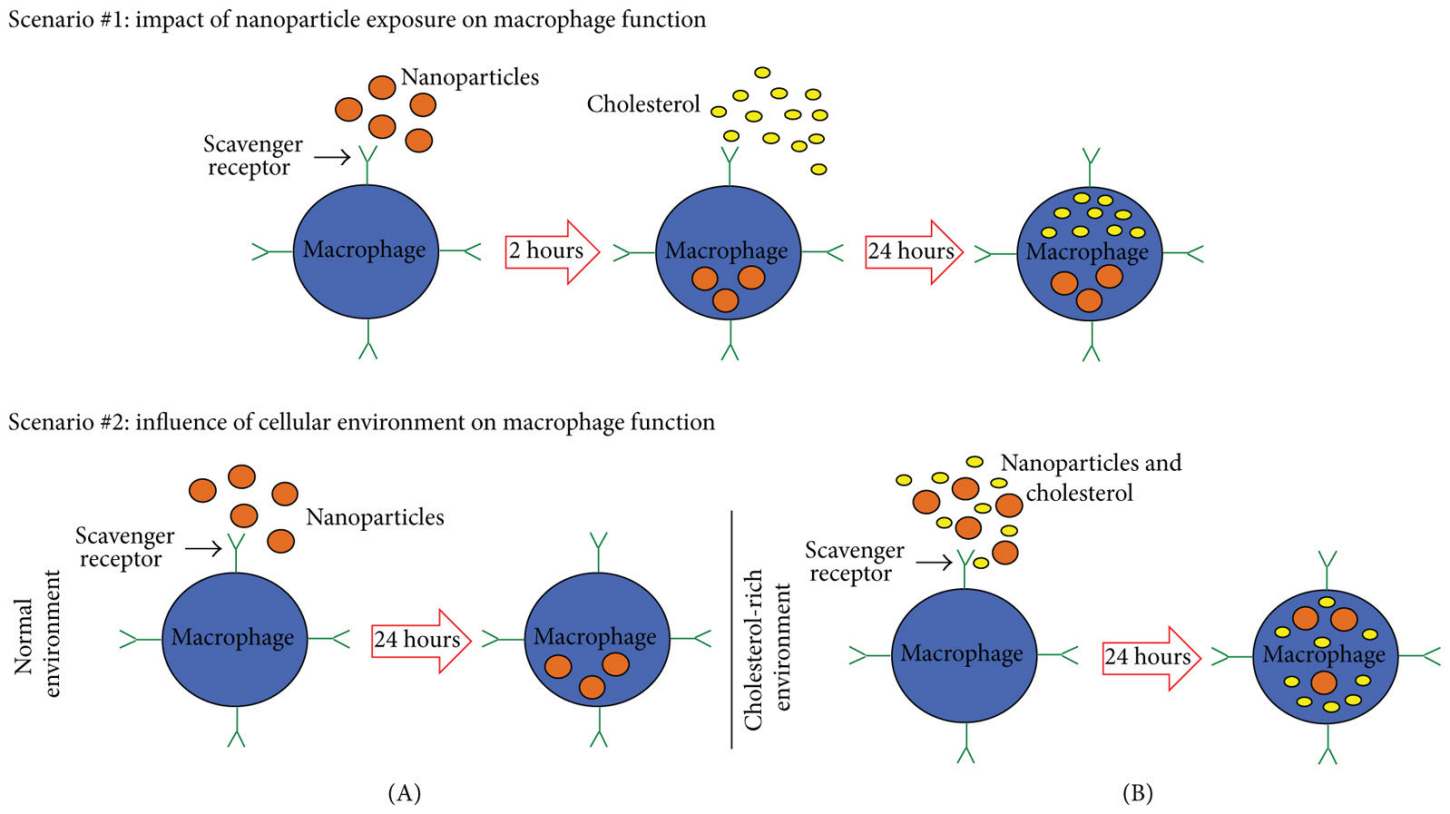
Overview of macrophage exposure scenarios. Scenario #1 investigates the impact of NP exposure on macrophage function. Macrophages were exposed for 2 h to 20 nm AgNPs, 110 nm AgNPs, or 20 nm Fe_3_O_4_ NPs. Media containing NPs were then removed and cells were treated with cholesterol (20 *μ*g/mL) for 24 h. Alterations in toxicity and macrophage function (cholesterol uptake) were assessed. Scenario #2 examined the influence of the cellular environment on macrophage function during an exposure to NPs. This scenario included (A) an environment without cholesterol present and (B) an environment with cholesterol present. Macrophages were exposed for 24 h to 20 nm AgNPs, 110 nm AgNPs, or 20 nm Fe_3_O_4_ NPs for 24 h in (A) serum-free media or (B) serum-free media containing cholesterol (20 *μ*g/mL). Alterations in macrophage toxicity and function were then assessed.

Based on the need for studies examining how exposure to NPs influences macrophage function and understanding alterations in biological responses to NPs in different cellular environments, we utilized two *in vitro* exposure scenarios (Figure 1). Scenario #1 was designed to evaluate the impact of NP exposure on macrophage function. In these experiments, macrophages were exposed to NPs (20 nm AgNPs, 110 nm AgNPs, or 20 nm Fe_3_O_4_ NPs) and then treated with cholesterol to assess alterations in cholesterol uptake. Scenario #2 was designed to investigate how different cellular environments influence macrophage responses to NPs. In these experiments, macrophages were exposed to NPs in either serum-free media or serum-free media containing high levels of cholesterol. Lastly, these studies evaluated the role of SR-B1, a receptor known to be involved in macrophage responses to both NPs and cholesterol.

## 2. Materials and Methods

### 2.1. NP Characterization

20 nm and 110 nm AgNPs suspended in citrate and 20 nm Fe_3_O_4_ NPs suspended in PVP were procured from NanoComposix (San Diego, CA) at a concentration of 1 mg/mL. The hydrodynamic size and zeta potentials (ZetaSizer Nano-ZS, Malvern) were characterized in DI water with NPs at a concentration of 25 *μ*g/mL (*n* = 3/particle). Another set of NPs (25 *μ*g/mL) were incubated for 24 h in cholesterol (20 *μ*g/mL) and assessed for hydrodynamic size and zeta potential following a series of centrifugations and washes with deionized water. The concentrations of NPs evaluated were chosen due to the use of these concentrations in our previous studies and the work of others [12, 25, 26]. The cholesterol concentration utilized was based on the manufacturer’s instructions for the measurements of cholesterol uptake (Cayman Chemical Company, Ann Arbor, MI). NPs were further characterized by hyperspectral darkfield microscopy (Cytoviva, Auburn, AL). Bare NPs or NPs incubated for 24 h in cholesterol (20 *μ*g/mL) were loaded onto premium clean microscope slides and mean spectrums were created utilizing pixels with an intensity of 1000 or greater. Mean spectrums were then compared between bare NPs and NPs incubated in cholesterol for the assessment of alterations in NP spectra indicative of cholesterol coating or formation of a NP biocorona.

### 2.2. Cell Culture

Mouse macrophages (RAW264.7) (ATCC, Manassas, VA) were cultured in Dulbecco’s modified eagle media (DMEM) containing 10% FBS and maintained in flasks under standard conditions of 37°C and 5% CO_2_. All experiments were performed at 90% confluency and in serum-free media conditions in order to inhibit protein-NP interactions resulting in protein corona formation.

### 2.3. Cytotoxicity

Macrophages were grown to 90% confluency in 96-well plates (Costar) and exposed to 20 nm AgNPs, 110 nm AgNPs, or 20 nm Fe_3_O_4_ NPs at concentrations of 6.25 12.5, 25, or 50 *μ*g/mL for 2h or 24h. The concentration range evaluated for cytotoxicity was selected based on previous *in vitro* experimentation of NPs [12, 25]. Changes in cell viability were assessed using the MTS assay (Promega, Madison, WI) via manufacturer’s instructions using a spectrophotometer (BioTek Synergy HT, BioTek, Winooski, VT). A NP concentration of 25 *μ*g/mL was selected for subsequent experiments due to limited induction of cytotoxicity at this concentration.

Macrophages were grown to 90% confluency in 96-well plates (Costar) and exposed to 20 nm AgNPs, 110 nm AgNPs, or 20 nm Fe_3_O_4_ NPs at a concentration of 25 *μ*g/mL in serum-free media for 2 h and then treated with cholesterol (20 *μ*g/mL) for 24 h. In a separate set of experiments macrophages were exposed to 20 nm AgNPs, 110 nm AgNPs, or 20 nm Fe_3_O_4_ NPs at a concentration of 25 *μ*g/mL in serum-free media containing cholesterol (20 *μ*g/mL) or without cholesterol present for 24 h. Changes in cell viability were again assessed using the MTS assay (Promega, Madison, WI) via manufacturer’s instructions using a spectrophotometer (BioTek Synergy HT, BioTek, Winooski, VT).

### 2.4. Macrophage Uptake of NPs

Macrophages were grown to 90% confluency in 24-well plates (Costar) or microscope chamber slides. Macrophages were exposed for 2 h to 20 nm AgNPs, 110 nm AgNPs, or 20 nm Fe_3_O_4_ NPs at a concentration of 25 *μ*g/mL in serum-free media. In a separate set of experiments macrophages were exposed for 24 h to 20 nm AgNPs, 110 nm AgNPs, or 20 nm Fe_3_O_4_ NPs at a concentration of 25 *μ*g/mL in serum-free media with cholesterol (20 *μ*g/mL) or without. Following exposure cells in 24-well plates were washed with PBS and collected by detachment with 250 *μ*L of trypsin and neutralization with an equal volume of media. NP uptake was evaluated by alterations in side scatter shift through flow cytometry (Accuri C6 Flow Cytometer, BD Biosciences, San Jose, CA). Side scatter shift values were normalized to controls and expressed as a fold change. Following exposure, cells in microscope chamber slides were fixed with 2% paraformaldehyde. Darkfield microscopy (Cytoviva, Auburn, AL) was utilized to confirm NP uptake within macrophages.

### 2.5. Alterations in Cholesterol Uptake due to NP Exposure

Cholesterol uptake was measured utilizing a cholesterol uptake cell-based assay kit via manufacturer’s instructions (Cayman Chemical Company, Ann Arbor, MI). This kit uses fluorescently labeled cholesterol to assess cellular uptake of cholesterol. Macrophages were grown to 90% confluency in 96-well plates (Costar) and exposed to 20 nm AgNPs, 110 nm AgNPs, or 20 nm Fe_3_O_4_ NPs for 2 h in serum-free media. Following the 2 h exposure to NPs macrophages were treated with fluorescently labeled cholesterol at a concentration of 20 *μ*g/mL for 24 h or serum-free media without cholesterol present. In a separate set of experiments macrophages were exposed for 24 h to 20 nm AgNPs, 110 nm AgNPs, or 20 nm Fe_3_O_4_ NPs at a concentration of 25 *μ*g/mL in serum-free media with (20 *μ*g/mL) or without cholesterol present. Media were then removed and replaced with a cell assay buffer and read using a fluorescent plate reader (BioTek Synergy HT, BioTek, Winooski, VT) to measure cholesterol uptake via manufacturer’s instructions (Cayman Chemical, Ann Arbor, MI) and background fluorescence was subtracted. Cholesterol uptake was qualitatively confirmed by fluorescent microscopy (Nikon Eclipse TE 2000-E, Tokyo, Japan) in macrophages grown on microscope slides and exposed to 110 nm AgNPs with or without cholesterol present. Nuclei were stained with DAPI and visualized as blue whereas cholesterol was visualized as green.

### 2.6. NP-Induced Alterations in Scavenger Receptor-B1 Expression

Macrophages were grown to 90% confluency in 24-well plates (Costar) and exposed to 20 nm AgNPs, 110 nm AgNPs, or 20 nm Fe_3_O_4_ NPs for 2 h in serum-free media. In a separate set of experiments macrophages were exposed for 24 h to 20 nm AgNPs, 110 nm AgNPs, or 20 nm Fe_3_O_4_ NPs at a concentration of 25 *μ*g/mL in serum-free media with (20 *μ*g/mL) or without cholesterol present. Macrophages were washed with PBS and collected by detachment with 250 *μ*L of trypsin and neutralization with an equal volume of media. Macrophages were then treated with 2% paraformaldehyde and stained with a fluorescently labeled scavenger receptor-B1 (SR-B1) antibody (1:100) (NB400-104, Novus Biologicals, Littleton, CO). Following a series of washes macrophage surface expression of SR-B1 was evaluated by flow cytometry (Accuri C6 Flow Cytometer, BD Biosciences, San Jose, CA). The mean fluorescent signal from no stain controls was subtracted from SR-B1 stained samples to remove any background autofluorescence.

### 2.7. Statistical Test

A one-way ANOVA test was performed using Dunnett’s post hoc analysis where applicable to determine significant differences in the dataset (*p* < 0.05). All data is presented as mean ± standard error of means (*n* = 3–6/group).

## 3. Results and Discussion

### 3.1. NP Characterization

**Figure 2:**
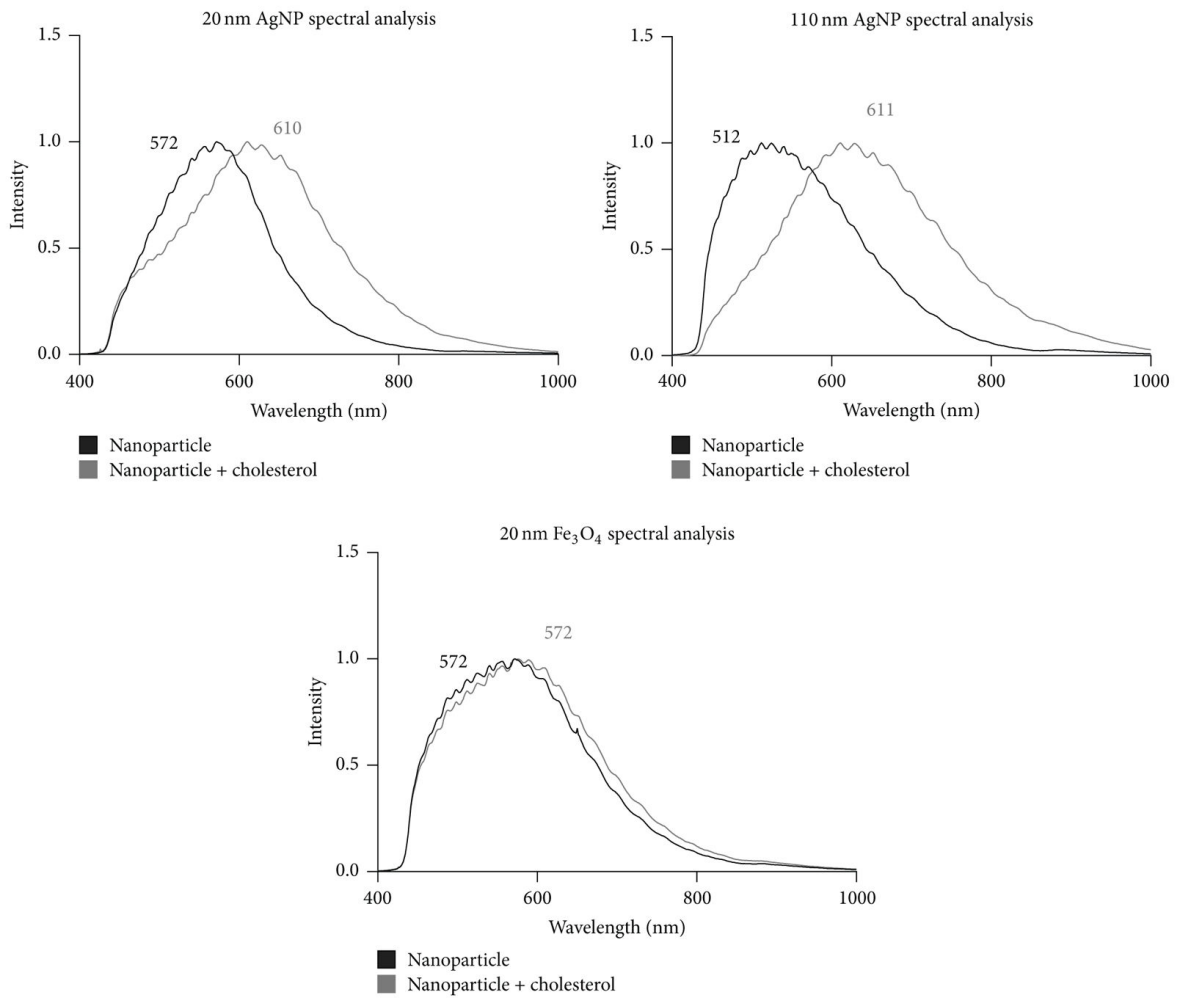
Hyperspectral profiles of 20 nm AgNPs, 110 nm AgNPs, and 20 nm Fe_3_O_4_ NPs in either water or cholesterol (20 *μ*g/mL). Following a 24 h incubation in water or cholesterol (20 *μ*g/mL) NPs were collected via centrifugation and underwent a series of washes. NPs were then loaded onto premium clean microscope slides and assessed by hyperspectral darkfield microscopy. NP spectra were created utilizing pixels with an intensity of greater than 1,000. Numbers represent the wavelength of the spectral peak; black denotes NPs in water whereas gray denotes NPs incubated with cholesterol.

Dynamic light scattering verified the sizes of procured NPs while all NPs demonstrated negative *ζ*-potentials (Table 1). Specifically, the citrate suspended AgNPs were determined to have a more negative *ζ*-potential as compared to the PVP suspended Fe_3_O_4_ NPs. Incubation with cholesterol (20 *μ*g/mL) for 24 h resulted in slight increases in hydrodynamic size as well as a reduction in *ζ*-potential for all NPs (Table 1). Hyperspectral analysis was performed on NPs to determine differences in spectra following 24 h incubation in cholesterol (Figure 2). A comparison of all NPs demonstrates differences in spectra that were likely based on NP identity, suspension material, and size. 20 nm AgNPs were red shifted compared to 110 nm AgNPs likely due to differences in size upon addition of cholesterol (Figure 2). 20 nm AgNPs and Fe_3_O_4_ NPs demonstrated similar spectral peaks at 572 nm; however Fe_3_O_4_ NPs exhibited a broader curve. The identical spectral peaks are likely due to both NPs having similar sizes (thus similar scattering) whereas the 20 nm AgNPs have a narrower peak due to their metallic nature. Incubation with cholesterol resulted in a red shift for the AgNPs indicative of association of cholesterol with the surface of the AgNPs (Figure 2). However the incubation of Fe_3_O_4_ NPs with cholesterol did not demonstrate any shifts in the spectral peak but exhibited a slight broadening of the spectrum.

The alterations we observed in hydrodynamic size, *ζ*-potential, and shifts in spectra are similar to changes we have seen in our previous work investigating the implications of biocorona on AgNP toxicity [12, 27]. The biocorona forms on NPs following their introduction in physiological environments as biomolecules interact and coat the NP surface [27, 28]. Specifically, we have demonstrated slight increases in hydrodynamic size, decreases in *ζ*-potential, and red shifts in spectra following addition of proteins such as albumin and high-density lipoprotein onto the surface of AgNPs [27]. This is likely occurring in our current study because cholesterol associates with the surface of the NPs. In these previous studies, we have also demonstrated that addition of these individual proteins can influence cell-NP interactions [12]. Further, in an assessment of proteins that bind to NPs following incubation in 10% fetal bovine serum, we have identified multiple apolipoproteins that ubiquitously associate with AgNPs [27]. Based on this binding of apolipoprotein binding to AgNPs as previously reported, it was expected that the cholesterol utilized in our current study would also associate with NPs. This biocorona formed following incubation in cholesterol is of interest for further study and has high human relevance. Specifically, individuals are known to have differing amounts of cholesterol within their circulation, which will influence the identity of the NP biocorona in terms of differential biocoronal cholesterol content. These alterations in cholesterol content of the biocorona will likely influence cell-NP interactions and toxicity on an individual basis due to the cholesterol content within the circulation.

### 3.2. Nanoparticle-Induced Cytotoxicity

**Figure 3:**
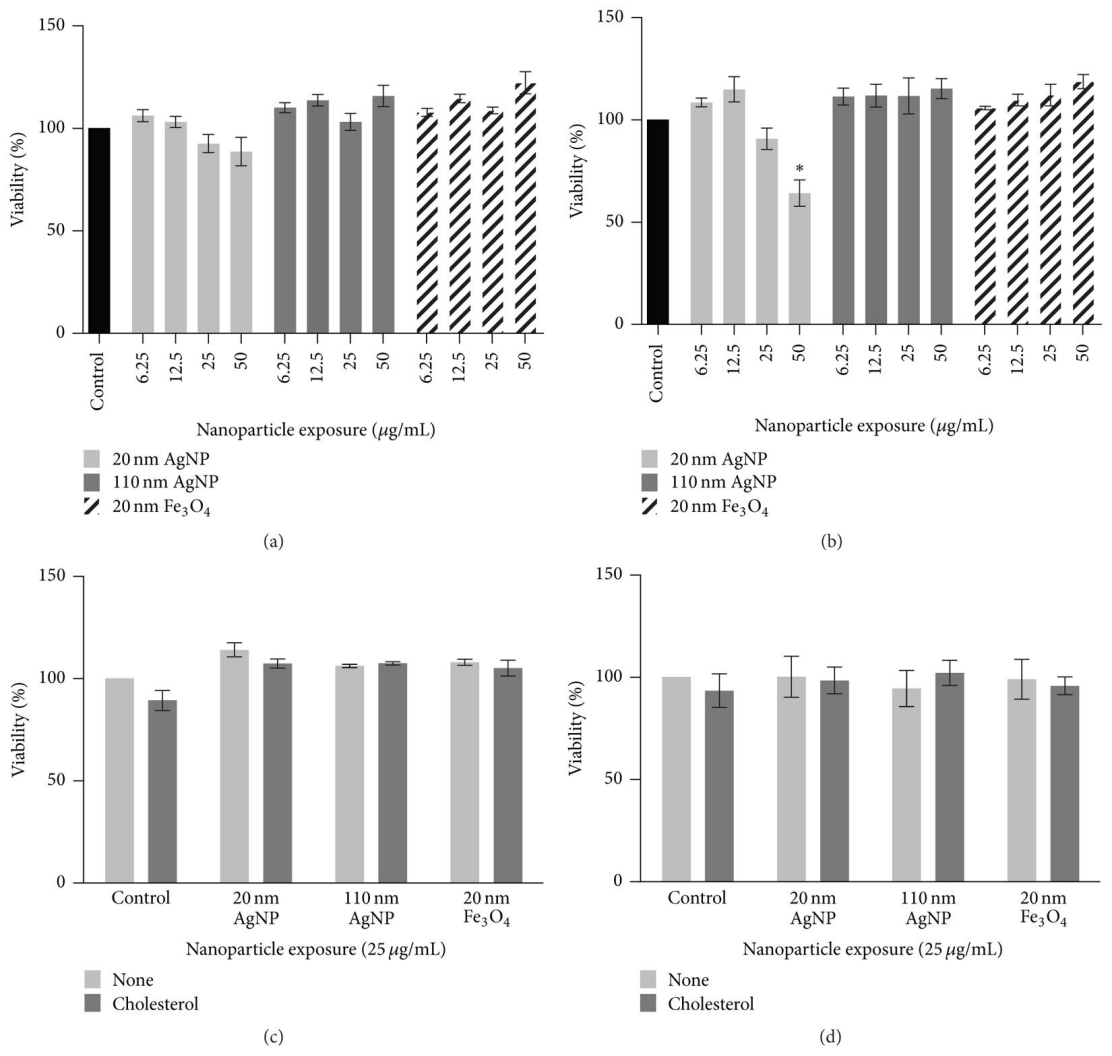
Cell viability changes in macrophages following exposure to 20 nm AgNPs, 110 nm AgNPs, or 20 nm Fe_3_O_4_ NPs at 6.25, 12.5, 25, or 50 *μ*g/mL for (a) 2 h or (b) 24 h. (c) Alterations in cell viability following a 2 h exposure to 20 nm AgNPs, 110 nm AgNPs, or 20 nm Fe_3_O_4_ NPs (25 *μ*g/mL) and a subsequent 24 h treatment to either serum-free media or serum-free media containing cholesterol (20 *μ*g/mL). (d) Cell viability following a 24h exposure to 20 nm AgNPs, 110 nm AgNPs, or 20 nm Fe_3_O_4_ NPs (25 *μ*g/mL) in either serum-free media or serum-free media containing cholesterol (20 *μ*g/mL). Values are expressed as mean ± SEM (*n* = 3–6/group). * indicates significant difference from controls (untreated) (*p* < 0.05).

A dose-response study was conducted on macrophages to determine a NP concentration for use in subsequent evaluation that did not induce overt cytotoxicity (Figure 3). No significant cytotoxicity was determined following a 2 h exposure to 20 nm AgNPs, 110 nm AgNPs, or 20 nm Fe_3_O_4_ NPs at concentrations of 6.25, 12.5, 25, or 50 *μ*g/mL (Figure 3(a)). Exposure to NPs at the same concentrations for 24 h was only found to induce significant cytotoxicity in macrophages exposed to 50 *μ*g/mL of 20 nm AgNPs (Figure 3(b)). Based on this cytotoxicity data, a NP concentration of 25 *μ*g/mL was utilized for all subsequent experiments, as it did not induce overt cytotoxicity.

In comparison to our previous work across the same range of concentrations in rat lung epithelial cells and rat aortic endothelial cells, the mouse macrophages used in this study are less susceptible to AgNP-induced cytotoxicity [12]. Specifically, previous studies revealed that rat aortic endothelial cells demonstrated significant cytotoxicity when exposed to 25 *μ*g/mL of 20 nm AgNP at 3 h. Further, both rat lung epithelial cells and rat aortic endothelial cells exhibited significant cytotoxicity at 6 h when exposed to 25 and 50 *μ*g/mL of 20 nm AgNPs, while rat aortic endothelial cells also demonstrated significant cytotoxicity at the concentration of 12.5 *μ*g/mL of 20 nm AgNPs. Based on these data from our current and previous work, there are cell specific differences in cytotoxicity in response to 20 nm AgNPs (macrophage < epithelial < endothelial). A critical implication of this finding is that conclusive assessments of NP toxicity cannot be gleaned from the investigation of cytotoxicity on one cell type, as they are variable in response. Cytotoxicity as an endpoint appears to be cell and NP specific therefore making broad generalizations regarding cytotoxicity inappropriate.

### 3.3. Impact of Cholesterol on Cytotoxicity

Macrophages were exposed to NPs at 25 *μ*g/mL for 2 h and, following exposure, NPs were removed and macrophages were treated for 24 h with either serum-free media containing cholesterol (20 *μ*g/mL) or without cholesterol (Figure 3(c)). Following this 24 h cholesterol treatment, cell viability was assessed. As observed before, none of the NPs were found to induce significant cytotoxicity at the 25 *μ*g/mL concentration (Figure 3(c)). Treatment with cholesterol did not induce cytotoxicity (Figure 3(c)). In a separate set of experiments macrophages were exposed to NPs (25 *μ*g/mL) in conjunction with cholesterol (20 *μ*g/mL) or in a cholesterol-free environment (serum-free media) for 24 h (Figure 3(d)). Following this coexposure, no differences were determined in the induction of cytotoxicity (Figure 3(d)). These results confirmed that there were no differences in cytotoxicity following cholesterol treatment and appropriate comparisons could be made in subsequent experiments investigating NP-induced alterations in macrophage function.

### 3.4. Macrophage Uptake of Nanoparticles

**Figure 4:**
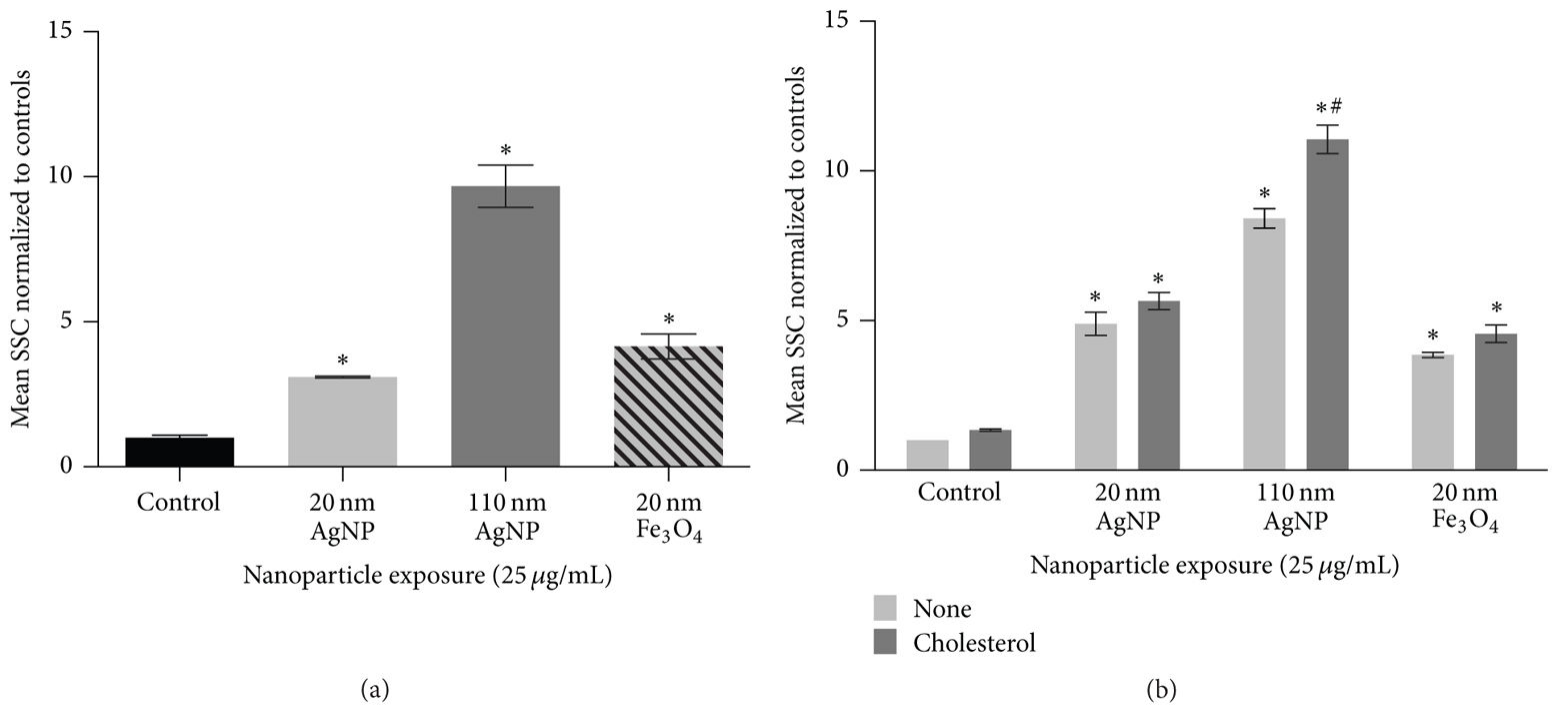
Measurement of NP uptake by assessment of changes in mean side scatter shift (SSC) via flow cytometry. (a) Macrophages were exposed to 20 nm AgNP, 110 nm AgNPs, or 20 nm Fe_3_O_4_ NPs at a concentration of 25 *μ*g/mL for 2 h and assessed for changes in side scatter shift (SSC). (b) Macrophages were exposed to 20 nm AgNP, 110 nm AgNPs, or 20 nm Fe_3_O_4_ NPs at a concentration of 25 *μ*g/mL for 24 h in either serum-free media or serum-free media containing cholesterol (20 *μ*g/mL). SSC values of macrophages exposed to NPs were normalized to control macrophages to produce a fold change. Values are expressed as mean ± SEM(*n* = 3/group). * indicates significant difference from controls (*p* < 0.05). # indicates significant difference from NP exposure in serum-free media (*p* < 0.05).

**Figure 5:**
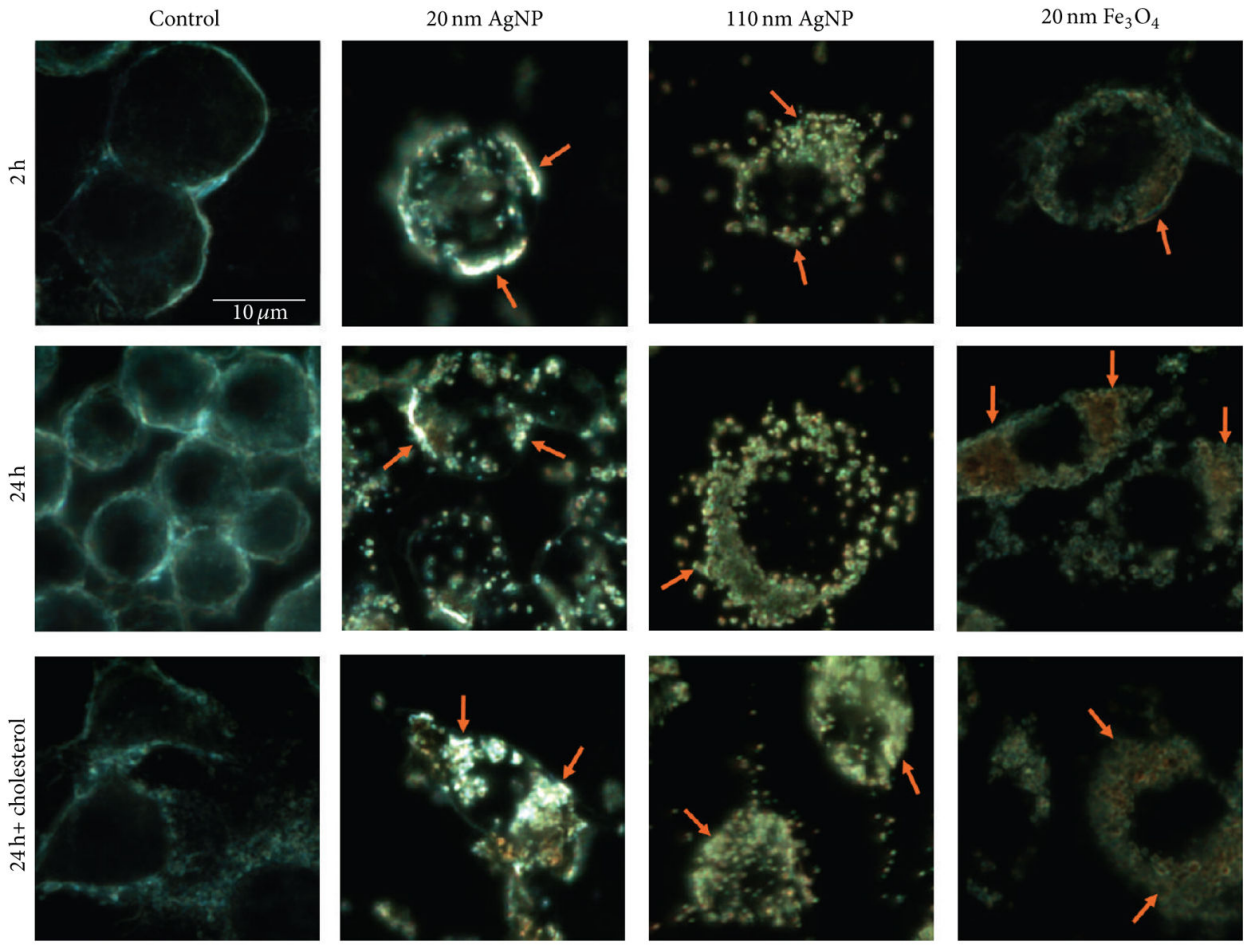
Representative enhanced darkfield images of macrophages exposed to 20 nm AgNPs, 110 nm AgNPs, or 20 nm Fe_3_O_4_ NPs visually demonstrating NP uptake. Images demonstrate macrophage uptake of NPs after 2 h and 24 h exposures in serum-free media or after a 24 h exposure in serum-free media containing cholesterol (20 *μ*g/mL). All images were taken at 100x magnification. Arrows indicate macrophage internalized NPs.

Uptake of NPs by macrophages was evaluated following a 2 h exposure to 20 nm AgNPs, 110 nm AgNPs, or 20 nm Fe_3_O_4_ NPs at a concentration of 25 *μ*g/mL (Figure 4(a)). To assess internalization of NPs, changes in mean side scatter of macrophages were measured by flow cytometry. Briefly, increases in mean side scatter correspond to increases in granularity of the cell indicative of NP internalization [12, 29, 30]. Following a 2 h exposure to each NP, mean side scatter was increased demonstrating the uptake of NPs by macrophages (Figure 4(a)). In an experiment designed to evaluate modifications in macrophage uptake of NPs in an environment with cholesterol present, macrophages were exposed to NPs (25 *μ*g/mL) in either serum-free media or serum-free media containing cholesterol (20 *μ*g/mL) for 24 h (Figure 4(b)). Exposure to NPs resulted in increased side scatter demonstrating uptake of each individual NP during the 24 h exposure (Figure 4(b)). When comparing the uptake following a 2 h exposure (Figure 4(a)) and a 24 h exposure (Figure 4(b)), similar changes in side scatter were observed. This demonstrates that the majority of uptake occurs within the first 2 h of an *in vitro* exposure. The cholesterol-rich environment resulted in increased uptake of 110 nm AgNP compared to the environment with cholesterol absent (Figure 4(b)). The cholesterol-rich environment however was not found to alter macrophage uptake of the 20 nm AgNPs or Fe_3_O_4_ NPs (Figure 4(b)). Uptake of NPs by macrophages was visually confirmed via enhanced darkfield microscopy (Figure 5).

All NPs in our current study were readily internalized by macrophages. This internalization was expected as *in vivo* studies have demonstrated localization of NPs within macrophages [31]. Macrophage uptake of NPs appears to occur quickly as there are only slight differences in uptake between 2 h and 24 h. Since this measurement of uptake utilizes changes in macrophage granularity it is difficult to make comparisons of uptake differences between NPs of variable size. However since two of our chosen NPs were of similar size (20 nm AgNP and Fe_3_O_4_) they can be more easily compared. The two 20 nm NPs (Ag and Fe) were taken up similarly at both time points even though they differed in composition (Ag and Fe) and suspension material (citrate and PVP), suggesting that size is a determining factor in internalization. When macrophages were exposed to NPs in a cholesterol-rich environment, uptake was increased, reaching significance only for 110 nm AgNPs. This finding suggests that certain NPs in individuals with high cholesterol may be differentially biodistributed compared to individuals with low cholesterol. This also suggests increased interactions with macrophages, which may enhance clearance and stimulate more robust inflammatory responses.

### 3.5. Modifications in Macrophage Function

In this study, we evaluated two exposure scenarios to determine how NP exposures may influence macrophage function and the influence of the cellular environment (Figure 1). Macrophage function was assessed by analyzing differences in cholesterol uptake. The concentration of 25 *μ*g/mL NPs utilized for these experiments was not found to induce significant cytotoxicity (Figure 3); therefore any alterations in macrophage function are not due to decreases in macrophage viability or numbers.

### 3.6. Impact of NP Exposure on Macrophage Function

**Figure 6:**
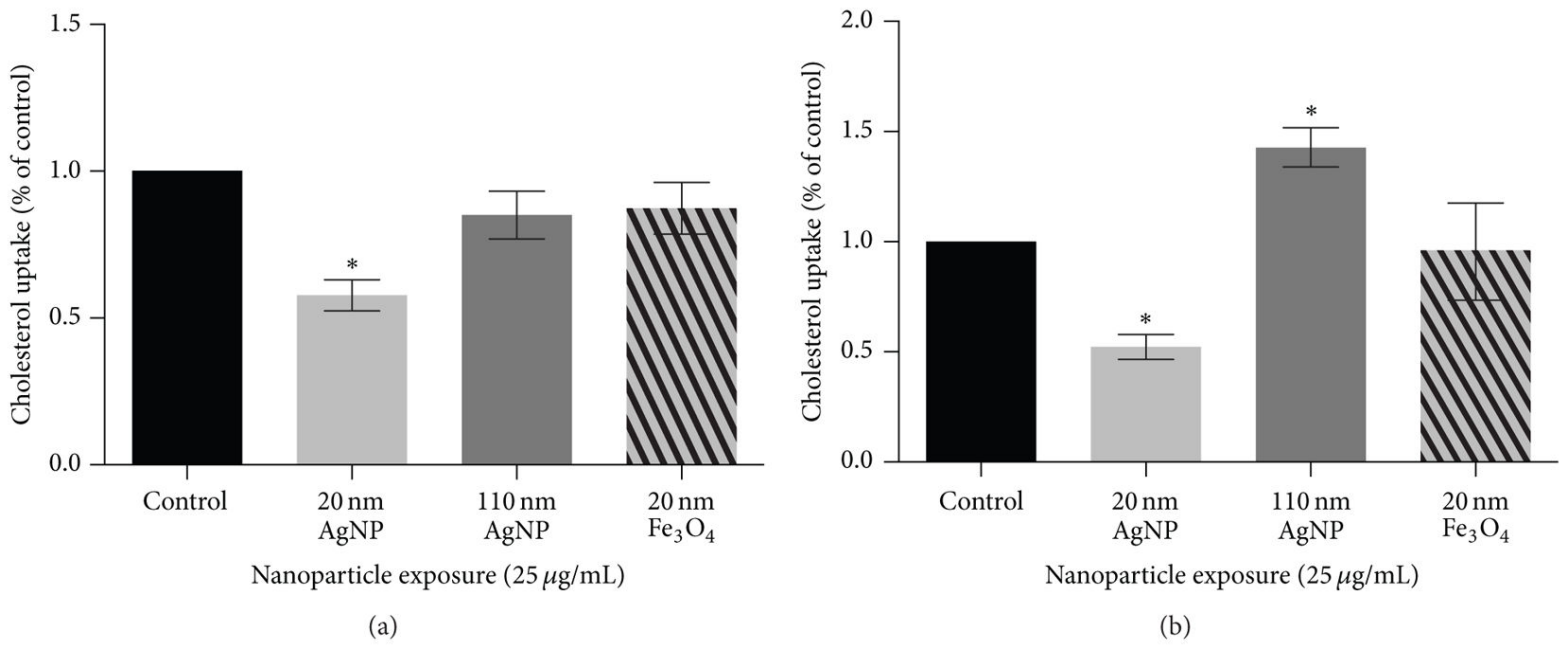
NP-induced alterations in macrophage uptake of cholesterol. (a) Macrophages were exposed to 20 nm AgNPs, 110 nm AgNPs, or 20 nm Fe_3_O_4_ NPs at a concentration of 25 *μ*g/mL for 2 h. NPs were removed prior to a 24 h treatment with fluorescently labeled cholesterol (20 *μ*g/mL) in serum-free media. (b) Macrophages were exposed to 20 nm AgNP, 110 nm AgNPs, or 20 nm Fe_3_O_4_ NPs at a concentration of 25 *μ*g/mL for 24 h in either serum-free media or serum-free media containing cholesterol (20 *μ*g/mL) and cholesterol uptake was measured at 24 h. Cholesterol uptake was measured via a spectrophotometer and normalized to control cholesterol uptake. Values are expressed as mean ± SEM (*n* = 3–8/group). * indicates significant difference from controls (*p* < 0.05).

In our first exposure scenario (Figure 1), macrophages were exposed to NPs (25 *μ*g/mL) for 2 h followed by measurement of cholesterol uptake (20 *μ*g/mL). Following the 24h cholesterol treatment, alterations in cholesterol uptake were assessed (Figure 6(a)). Exposure for 2 h to 20 nm AgNPs was found to reduce uptake of cholesterol compared to control (Figure 6(a)). No alterations in cholesterol uptake were demonstrated following a 2 h exposure to 110 nm AgNPs or 20 nm Fe_3_O_4_. These NP-induced modifications in cholesterol uptake by macrophages are likely driven by a variety of physicochemical properties including size, suspension material, and/or charge. NP size and surface area have been shown to be important for interactions with cells. Specifically, it has been shown in the study of NP immune cell interactions using mast cells that 20 nm AgNPs induce degranulation whereas 110 nm AgNPs do not [22]. In our current study, we utilized two AgNPs suspended in citrate while the Fe_3_O_4_ NPs were suspended in PVP. Interestingly, in our current study, cholesterol uptake was reduced following exposure to 20 nm AgNPs whereas no changes were exhibited following exposure to 20 nm Fe_3_O_4_ NPs. Although these NPs are of similar size they do differ based on suspension material and charge, which may alter NP-cell interactions.

### 3.7. Influence of Cellular Environment on Macrophage Function

**Figure 7:**
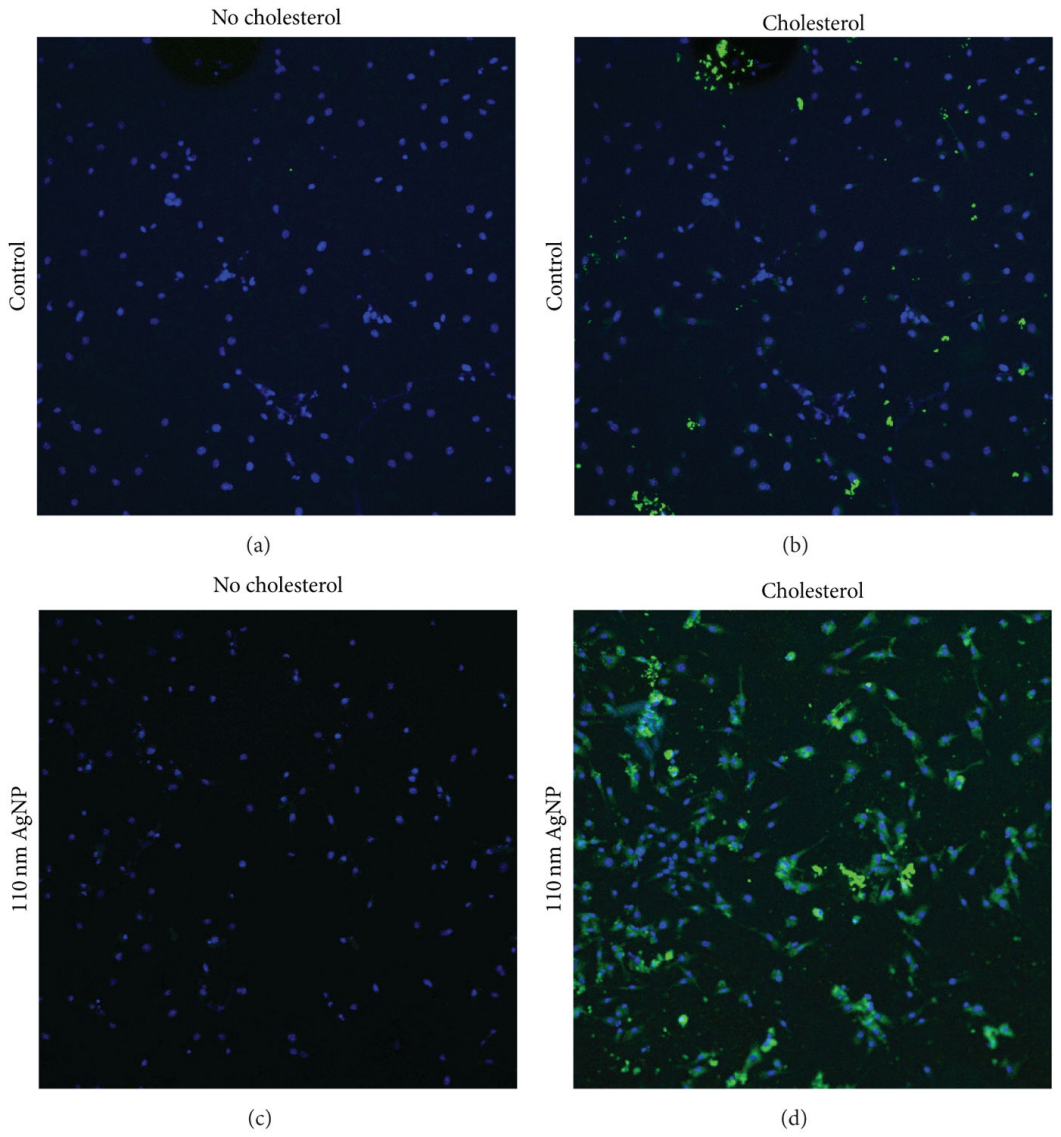
Confocal microscopy imaging of alterations in cholesterol uptake by macrophages exposed to NPs. (a) Control macrophages cultured in serum-free media for 24 h. (b) Macrophages cultured for 24 h in serum-free media containing cholesterol (20 *μ*g/mL). (c) Macrophages exposed for 24 h to 110 nm AgNPs (25 *μ*g/mL) in serum-free media. (d) Macrophages exposed for 24 h to 110 nm AgNPs (25 *μ*g/mL) in serum-free media containing cholesterol (20 *μ*g/mL). Blue represents DAPI stained nuclei whereas green areas represent fluorescently labeled cholesterol. All images were taken at 40x magnification with the confocal and detection parameters held constant between images.

In our second exposure scenario (Figure 1), macrophages were exposed to NPs (20 nm AgNPs, 110 nm AgNPs, or 20 nm Fe_3_O_4_ NPs) at 25 *μ*g/mL in serum-free media or serum-free media with cholesterol (20 *μ*g/mL) for 24 h (Figure 6(b)). Macrophages exposed to 20 nm AgNPs with cholesterol present for 24 h demonstrated a decrease in cholesterol uptake whereas exposure to 110 nm AgNPs caused an increase in cholesterol uptake (Figure 6(b)). Since 20 nm and 110 nm AgNPs were found to have different effects, this suggests that size is important in modifying cholesterol uptake in cholesterol-rich environments. It is possible that 110 nm AgNPs increase cholesterol uptake by acting as a carrier for cholesterol into the cell. Based on our previous research we have demonstrated that NPs of different sizes can result in differential association of proteins [27]. It is likely that 110 nm AgNPs bind substantially more cholesterol onto their surfaces thereby increasing macrophage cholesterol content following internalization of 110 nm AgNPs. Cholesterol uptake as compared to controls was not modified following exposure to 20 nm Fe_3_O_4_ NPs in serum-free media with cholesterol (Figure 6(b)). This finding suggests that Fe_3_O_4_ NPs may be useful for clinical applications, as it does not modify macrophage function in either the absence or presence of cholesterol. The increase in cholesterol uptake that occurred with 110 nm AgNP in serum-free media with cholesterol was visually confirmed via fluorescent microscopy (Figure 7). In an attempt to begin to understand the mechanism behind these responses we evaluated the receptor content on the surface of macrophages of the scavenger receptor-B1.

### 3.8. Nanoparticle-Induced Alterations in Macrophage Expression of Scavenger Receptor-B1

**Figure 8:**
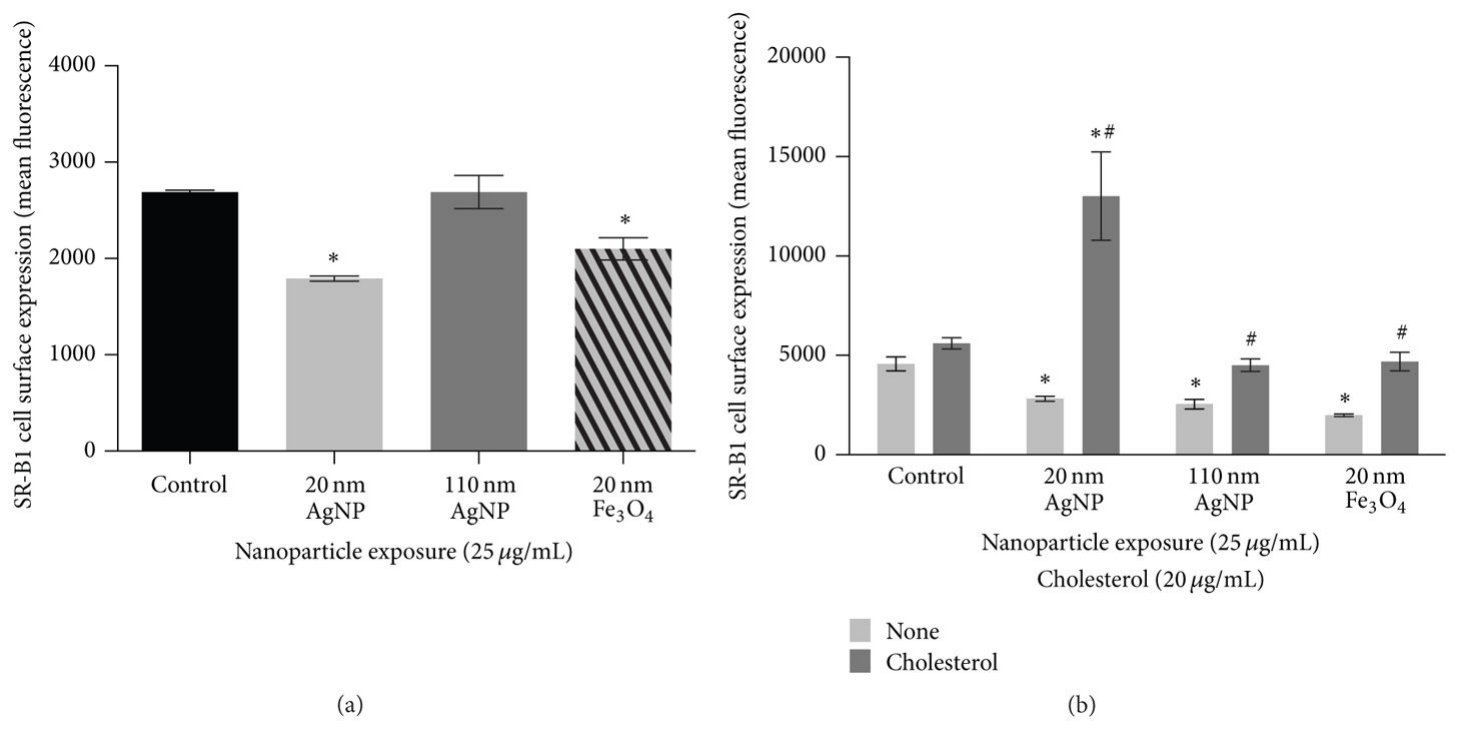
Alteration in macrophage surface receptor expression of scavenger receptor-B1 (SR-B1). (a) Macrophages were exposed to 20 nm AgNP, 110 nm AgNPs, or 20 nm Fe_3_O_4_ NPs at a concentration of 25 *μ*g/mL for 2 h and assessed for changes in SR-B1 cell surface expression by flow cytometry. (b) Macrophages were exposed to 20 nm AgNP, 110 nm AgNPs, or 20 nm Fe_3_O_4_ NPs at a concentration of 25 *μ*g/mL for 24 h in either serum-free media or serum-free media containing cholesterol (20 *μ*g/mL) and assessed for changes in SR-B1 cell surface expression. The mean fluorescent signal from no stain controls was subtracted from SR-B1 stained samples to correct for any background autofluorescence. Values are expressed as mean ± SEM(*n* = 3/group). * indicates significant difference from controls (*p* < 0.05). # indicates significant difference from NP exposure in serum-free media (*p* < 0.05).

Following a 2 h exposure to NPs (25 *μ*g/mL), macrophage cell surface expression of scavenger receptor-B1 (SR-B1) was analyzed via flow cytometry (Figure 8(a)). SR-B1 is involved in the uptake of both NPs and cholesterol by macrophages [18, 32]. Therefore, it is likely that NP exposure may alter the expression of SR-B1 on the surface of macrophages thereby modifying macrophage responses to cholesterol. Exposure to 20 nm AgNPs and Fe_3_O_4_ NPs was found to decrease SR-B1 expression on the surface of macrophages whereas 110 nm AgNP exposure was not found to modify expression as compared to controls (Figure 8(a)). Specifically, a 2 h exposure to 20 nm AgNPs was found to reduce SR-B1 receptor expression more so than other NPs evaluated. This decrease in SR-B1 receptor expression (Figure 8(a)) likely contributes to the decrease in subsequent cholesterol uptake as observed in Figure 6(a). Fe_3_O_4_ NPs were also found to reduce SR-B1 expression but, however, were not found to alter cholesterol uptake compared to control. It is likely that 20 nm AgNPs more readily interact with SR-B1 and have a higher affinity for the receptor due to their more negative charge. This higher affinity may not only reduce receptor expression but may also antagonize subsequent cholesterol binding with the receptor. Previous research has demonstrated that amphiphilic as well as 20 nm ZnO and 20 nm TiO_2_ NPs can reduce the expression of Class A scavenger receptors on cell surfaces [33, 34]. Further, amphiphilic NPs were also determined to competitively inhibit binding of oxidized low-density lipoprotein through NP-receptor interactions [33]. Our findings support these studies and demonstrate that NPs can also modulate surface expression of Class B scavenger receptors.

In addition to interactions with lipoproteins and negatively charged molecules/particles scavenger receptors are also known to interact and facilitate the removal of pathogens. Specifically, SR-B1^−/−^ mice infected with *Mycobacterium tuberculosis* demonstrated significant reductions in TNF*α*, IFN_γ_, and IL-10 as compared to wild-type mice [35]. This NP-induced reduction in SR-B1 expression seen in our current study may inhibit the immune response to subsequent microbial exposures due to decreased macrophage cell surface expression of SR-B1. Cell surface expression of SR-B1 was also evaluated following a 24 h exposure to NPs in either serum-free media or serum-free media with cholesterol (20 *μ*g/mL) (Figure 8(b)). All NPs were found to reduce cell surface receptor expression of SR-B1 as compared to controls following the 24 h exposure (Figure 8(b)). This demonstrates that prolonged exposure to NPs may reduce SR-B1 expression on the surface of macrophages and alter subsequent immune responses mediated via macrophages as well as normal macrophage function. This reduction may also limit the macrophage’s ability to clear successive exposure to other foreign particles or pathogens that are normally cleared via SR-B1. Previous research has demonstrated that exposure to Fe_3_O_4_ NPs for 24 h, at a concentration that did not cause cytotoxicity or an inflammatory response, reduced the phagocytic activity of macrophages following treatment with *Streptococcus pneumonia* [36]. Further macrophages exposed to Fe_3_O_4_ NPs were found to have suppressed induction of the IL-10 pathway, enhanced TNF-a production, and an inhibition of the transition from an M1- to M2- like activation state in response to *Streptococcus pneumonia* treatment. This reduced response to *Streptococcus pneumonia* was hypothesized to be due to Fe_3_O_4_ NP-induced alterations in scavenger receptor expression. In the presence of cholesterol 20 nm AgNPs increased cell surface expression of SRB1 (Figure 8(b)). This increased expression of SR-B1 suggests that individuals with high cholesterol may respond differently to NP exposures. Further this increased expression of SRB1 may also result in exacerbated inflammatory responses to secondary exposures. The presence of cholesterol however inhibited the reduction of SR-B1 expression observed when macrophages were exposed to NPs alone (Figure 8(b)). Taken together the cholesterol-rich environment alters the macrophage response to NPs in terms of phenotypic expression of SR-B1.

## 4. Conclusions

Overall this study demonstrates that macrophage function, as assessed by alterations in cholesterol uptake, is modified following NP exposures. Further, our research demonstrates that these modifications in macrophage function are not uniform and likely are dictated by various NP characteristics. For example, exposure to 20 nm AgNPs resulted in decreased macrophage uptake of cholesterol compared to 20 nm Fe_3_O_4_ NPs, which did not alter macrophage function in a cholesterol-rich environment. This finding demonstrates that modulation of macrophage function is not solely driven by NP size. Although NP exposure may not result in overt cytotoxicity, NPs may cause modifications in the normal function of key cell types such as macrophages. These modifications in function may influence disease progression, biodistribution of nanomedicines, and cellular responses to subsequent exposures.

## Figures and Tables

**Table 1:** Hydrodynamic size and zeta potential of NPs suspended in water or cholesterol.

Nanoparticle	Suspended in water		Suspended in cholesterol (20 *μ*g/mL)	
	Hydrodynamic size (nm)	Zeta potential (mV)	Hydrodynamic size (nm)	Zeta potential (mV)
20 nm AgNP	29.3 ± 0.2	−54.7 ± 0.7	31.1 ± 0.2	−50.0 ± 0.4
110 nm AgNP	106.7 ± 0.3	−61.2 ± 1.1	110.6 ± 0.6	−58.4 ± 0.3
20 nm Fe_3_O_4_	37.72 ± 0.1	−43.8 ± 1.2	40.23 ± 0.3	−38.0 ± 0.8
